# Fetal Doppler Evaluation to Predict NEC Development

**DOI:** 10.3390/jpm12071042

**Published:** 2022-06-25

**Authors:** Miriam Duci, Erich Cosmi, Pierpaolo Zorzato, Ambrogio Pietro Londero, Giovanna Verlato, Eugenio Baraldi, Eugenio Ragazzi, Francesco Fascetti Leon, Silvia Visentin

**Affiliations:** 1 Department of Women’s and Children’s, Division of Paediatric Surgery, University of Padua, 35100 Padua, Italy; ducimiriam@gmail.com (M.D.); francesco.fascettileon@unipd.it (F.F.L.); 2 Maternal Fetal Medicine Unit, Department of Women’s and Children’s, School of Medicine, University of Padua, 35100 Padua, Italy; pierpaolo.zorzato@gmail.com (P.Z.); silvia.visentin@unipd.it (S.V.); 3 Academic Unit of Obstetrics and Gynecology, Department of Neuroscience, Rehabilitation, Ophthalmology, Genetics, Maternal and Infant Health, University of Genoa, 16132 Genova, Italy; ambrogio.londero@gmail.com or; 4 Department of Women’s and Children’s, Division on Neonatal Intensive Care Unit, University of Padua, 35100 Padua, Italy; verlatogiovanna@gmail.com (G.V.); eugenio.baraldi@unipd.it (E.B.); 5 Department of Pharmaceutical and Pharmacological Sciences, University of Padua, 35100 Padua, Italy; eugenio.ragazzi@unipd.it

**Keywords:** necrotizing enterocolitis, Doppler flow velocimetry, fetal growth restriction, premature, predictive values

## Abstract

Antenatal factors play a role in NEC pathogenesis. This study aimed to investigate the predictive value of fetal ductus venosus doppler (DV) for NEC in fetal growth restriction fetuses (FGRF) and to assess the predictive accuracy of IG21 and Fenton curves in NEC development. Data from FGRF, postnatal findings, and Doppler characteristics were collected between 2010 and 2020 at a single center. Patients were then divided into two groups (i.e., with and without NEC). Bivariate and multivariate analyses were performed. We identified 24 cases and 30 controls. Absent or reversed end-diastolic flow (AREDF) and increased resistance in the DV were more impaired in cases (*p* < 0.05). Although the median birthweight was not different, the Fenton z-score was lower in NEC (*p* < 0.05). Fetal cardiopulmonary resuscitation, synchronized intermittent mandatory ventilation, neonatal respiratory distress, persistent patent ductus arteriosus (PDA), and inotropic support were more frequent in the NEC group. Furthermore, NEC patients had lower white blood cells (WBC) (*p* < 0.05). The predictive model for NEC (model 4), including Fenton z-score, WBC, PDA, and DV had an AUC of 84%. Fetal Doppler findings proved effective in predicting NEC in FGR. The Fenton z-score was the most predictive factor considering the fetal growth assessment showing high sensitivity.

## 1. Introduction

Fetal growth restriction (FGR) is an abnormal intrauterine growth pattern associated with higher perinatal mortality, newborn postnatal complications, and long-term sequelae in adulthood [1].

The severity of neonatal morbidity depends on the newborn’s prematurity, birthweight (BW), and the presence of fetal-maternal Doppler anomalies [2]. A higher umbilical artery pulsatility index (PI) and an absent or reversed end-diastolic flow (AREDF) in FGR are associated with poor neonatal outcomes. Currently, the timing of delivery in FGR is based on abnormal cardiotocography and ductus venosus (DV) pulsatility [3,4].

Necrotizing enterocolitis (NEC) is a devastating disease affecting around 1–5% of preterm newborns in neonatal intensive care units (NICU) and it is associated with a mortality ranging from 20% to 50% [5]. Its pathogenesis is multifactorial, involving vascular distribution, an immature intestinal barrier, altered innate and adaptive host immune responses, and the intestinal microbiome [6]. Observational studies suggest that NEC becomes more likely when prematurity is associated with FGR [6]. Despite advances in perinatal care, no factors clearly predict which newborns are more likely to develop NEC [6]. Postnatal algorithms taking into consideration clinical, instrumental, and biochemical variables have been proposed. However, available data on the predictive value of antenatal factors are sparse and controversial [7]. If antenatal factors could help identify newborns at a high risk of NEC, the disease could be diagnosed and treated more promptly.

Authors focusing on FGR previously found Doppler anomalies implicated in the pathogenesis of NEC, while recent studies have identified DV anomalies as an indicator of fetal heart failure and neonatal morbidity [4,8].

This study examined the role of DV in predicting NEC in FGR newborns. Furthermore, the value of the Fenton and INTERGROWTH-21st (IG21) curves in predicting which newborn with FGR would develop NEC was investigated.

## 2. Materials and Methods

This retrospective study was conducted in the Prenatal Diagnostics Section of a tertiary center. The study sample included preterm infants with FGR admitted to the NICU with or without NEC whose antenatal data were available. The exclusion criteria were as follows: preterm newborn without FGR; maternal or neonatal infections; genetic, chromosomal, or structural anomalies confirmed at birth and by karyotype analysis; outborn preterm newborn; newborn dying within the first two days of life; and no prenatal estimated fetal weight or Doppler findings. Premature FGR newborns without diagnosed NEC who matched with the cases for gestational age (GA), served as the controls.

Prematurity was defined as birth between 23 and 36 + 6 weeks of gestation, following spontaneous labor or for iatrogenic reasons. GA was confirmed via routine ultrasound examination in the first trimester. FGR was defined according to the New Delphi Consensus [9]. The estimated fetal weight percentile was based on the Hadlock C curve [10]. A BW below the 10th percentile confirmed FGR [11,12].

FGR was clinically managed during gestation following international guidelines, with weekly Doppler testing for fetal well-being, fortnightly fetal biometric measurements, and cardiotocography [3]. Fetal and maternal vessel sampling followed the International Society of Ultrasound in Obstetrics and Gynecology guidelines [2]. Maternal Doppler velocimetry of the uterine arteries in bilateral protodiastolic (notch) incision cases were recorded retrospectively. The mean uterine artery resistance index was calculated from the average indices of three consecutive waveforms for both arteries. The umbilical artery PI was considered pathological if it is above the 95th percentile for GA, with AREDF [2]. The definition of brain sparing was based on the PI of the middle cerebral artery or a cerebral/placental ratio below the 5th percentile for GA [2]. DV pulsatility was considered pathological if the PI was above the 95th percentile for GA or if there was no a-wave [2].

All patients underwent repeat testing, and statistical analysis was performed on the final findings. The delivery timing was dependent on BW percentile, fetal and maternal Doppler, cardiotocography, and the mother’s obstetric condition (e.g., hypertensive disorders).

The following data were recorded in each case:

- Mother’s age, parity, obstetric history, preeclampsia or gestational diabetes, suspected or known chorioamnionitis, placental insufficiency, and premature rupture of membranes [13,14,15,16,17];
- At delivery: GA; mode of delivery; reason for cesarean section; newborn’s sex and BW; Apgar score at 5 and 10 min; need for major or minor neonatal resuscitation;
- On NICU admission: neonatal C-reactive protein, white blood cells (WBC) count, serum pH, hemoglobin, and platelet count.

The recorded short-term outcomes recorded were: respiratory distress syndrome (RDS), respiratory acidosis, need for ventilatory support within the first 24 h of life, administration of surfactant, need for inotropic support, severe intraventricular cerebral hemorrhage (grades 3–4), sepsis, recurrent apneas, persistent patent ductus arteriosus (PDA), patent foramen ovale (PFO), and the onset of NEC.

**Figure 1:**
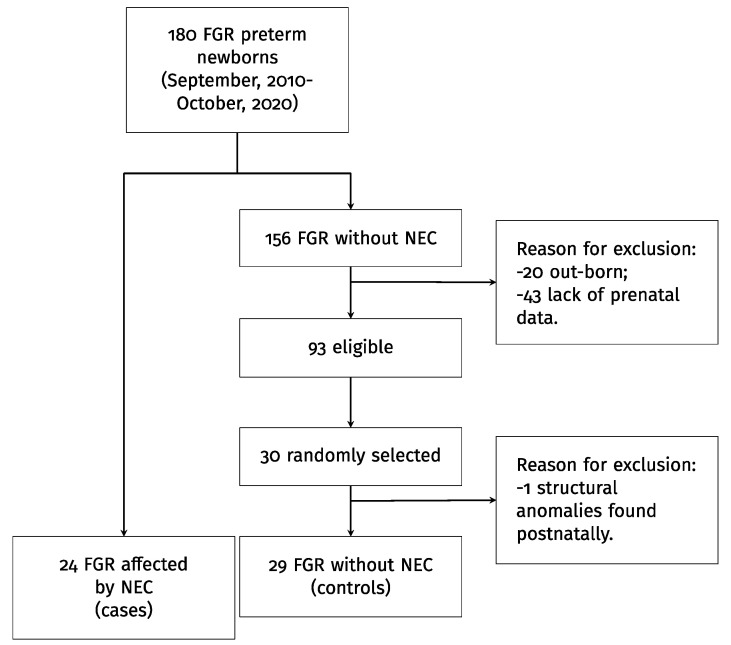
Flowchart.

NEC was diagnosed on a clinical and radiological basis (≥Bell’s stage I), by visual inspection at laparotomy and/or histological evidence. Treatment was medical or surgical, and was based on a multidisciplinary approach [18]. All data were collected in an Excel spreadsheet. NEC diagnosis was found in 24 FGR fetuses (Figure 1).

Twenty-five controls were needed to detect a large effect size (0.8) of the prevalence of abnormal fetal Doppler findings in cases with/without NEC with a power of 80% and significance level of 0.05 [19]. A control group of 30 fetuses was randomly selected, given the risk of missing data. The controls had FGR, a median GA of 30.4 weeks, were born during the same period and had no evidence of NEC. One control was subsequently excluded after morphological anomalies were found postnatally (Figure 1).

The data were analyzed with R (version 3.6.3; R Foundation for Statistical Computing, Vienna, Austria, http://www.R-project.org/, accessed on 3 June 2022). A two-tailed *p*-value < 0.05 was considered statistically significant. The results are presented as medians and interquartile ranges (IQR) or means and standard deviations (±SD) for continuous variables, as absolute numbers and percentages for categorical data, and as ratios or values (e.g., odds ratio, etc.) and 95% confidence intervals (CI). Numerical variables were compared between groups using a nonparametric or parametric approach (Wilcoxon’s test or *t*-test, respectively), and categorical data used the chi-square or Fisher’s exact test. Univariate and multivariate logistic analyses were performed to determine the relationship between NEC and possible predictors. All independent factors with a *p* < 0.100 were included in the multivariate models, and a stepwise assessment was used to produce the final model. All potential interaction terms were considered in the multivariate models and were excluded if they were not significant. A prediction accuracy analysis was performed using receiver operator characteristic (ROC) curves. Sensitivity and specificity were assessed using the best ROC threshold, and areas under the curve (AUC) of different ROCs were compared using De Long’s test.

## 3. Results

### 3.1. Population

Table 1 shows the characteristics of cases and controls.

Fetal Doppler findings were more impaired in newborns who developed NEC, with a higher prevalence of cases of umbilical artery AREDF and a greater resistance in the DV than in controls (*p* < 0.05) (Table 1). No other differences were found between the groups.

Table 2 shows the newborns’ characteristics.

The z-scores obtained from the Fenton growth chart varied significantly between the cases and controls (*p* < 0.05). WBC counts obtained on admission to the NICU were significantly lower in NEC cases than in controls (*p* < 0.05). Furthermore, the need for fetal cardiopulmonary resuscitation or synchronized intermittent mandatory ventilation, the incidence of neonatal RDS or PDA, and the use of inotropic support just after birth also differed significantly between the two groups (Table 2).

### 3.2. Prenatal NEC Predictors

Table 1 and Table 3 show that AREDF in the umbilical artery and a high PI in the DV were more common among newborns with NEC than in controls. Moreover, a very high PI in the DV was the most significant and independent predictor of the onset of NEC. This emerged in model 1 (Table 4), where the AUC for an increased PI in the DV was 66% (95% CI 54–77%), the specificity was 90% (95% CI 79–100%), and the sensitivity was 42% (95% CI 21–62%) (Table 3).

### 3.3. Postnatal NEC Predictors

Table 3 shows the most significant postnatal predictors of the onset of NEC. In the univariate logistic regression models, the z-score for BW using the Fenton growth chart, the WBC on NICU admission, the need for cardiopulmonary resuscitation just after delivery, PDA, and the inotropic support use were all predictive factors. The best cut-off for the Fenton z-score was ≤−1.62, and for the WBC was ≤5255 × 10^9^/L (Table 3).

Table 4 provides details of the multivariate models.

In model 2 (Table 4), the Fenton z-score was a significant predictor of NEC, irrespective of BW. In model 3 (Table 4), concerning the best postnatal predictors, the AUC was 82% (95% CI 71–94%), with a sensitivity higher than the model’s specificity. For model 4 (Table 4), which includes prenatal Doppler findings and postnatal parameters, its accuracy in predicting NEC had an AUC of 84% (95% CI 72–95%), which was significantly greater than that of the other models (*p* < 0.05 for model 1; *p* < 0.05 for model 2; and *p* = 0.385 for model 3).

## 4. Discussion

### 4.1. Principal Findings and Results

Fetal Doppler findings have emerged as a significant predictor of NEC, and the strongest predictor in our study was the DV’s PI. The models based on fetal Doppler values were highly specific but showed low sensitivity in predicting NEC in FGR newborns. Among the other factors we considered, the most useful for predicting NEC were the Fenton z-score and the newborn’s WBC count on NICU arrival. Using the DV’s PI in multivariate models improved the specificity when predicting NEC in FGR newborns.

### 4.2. Clinical and Research Implications

NEC in preterm neonates is a disease that remains poorly understood and is associated with high morbidity and mortality rates [20]. Numerous efforts have been made to identify postnatal factors that predispose patients to developing NEC. However, little is known about antenatal factors. Despite improvements in our understanding of the main factors involved in its pathogenesis, we still lack a clear picture of this disease and how it can be prevented and treated [21].

We hypothesized that a combination of prenatal Doppler findings and postnatal risk factors would allow for prediction of NEC in FGR newborns.

Unlike some other studies, there were no differences regarding cortisone administration during pregnancy between the groups in our study [7]. A previous matched case-control study included all newborns affected by NEC and they were one-to-one control matched for GA and birth year; in contrast, the present study focused only on FGR newborns with available maternal and fetal Doppler data [22]. In contrast with the previous report, the main maternal disorders we considered in addition to chorioamnionitis did not emerge as predictors of NEC in FGR newborns [22]. Furthermore, different fetal growth curves were also compared to identify the best one for detecting newborns at a greater risk of NEC [11,12]. Our study confirmed the correlation between fetal Doppler anomalies and NEC that was previously reported in the literature [7,23,24]. Moreover, we found the venous compartment to be more predictive of the NEC onset. The link between the DV and NEC has on recently been studied in a small series [4,8,23]. When Baschat et al. examined the relationships between fetal and maternal Doppler parameters and NEC onset, their univariate analysis showed a correlation between Doppler anomalies (AREDF and abnormal venous indices) and NEC. However, it lacked multivariate analysis confirmation [8]. The only significant association with abnormal Doppler indices was for the DV and intrauterine death [8]. Similarly, Raboisson et al. investigated the role of prenatal Doppler characteristics in predicting NEC [24]. Their cohort of 12 newborns with FGR who developed this disease revealed a significant association between NEC and bilateral notching of the uterine arteries, uterine artery mean resistance index, aortic isthmus diastolic blood flow velocity integrals, and an absent or negative “a” wave for the DV. Furthermore, logistic regression analysis showed that bilateral uterine artery notching predicted NEC with 83.3% sensitivity and 70.3% specificity. Our cohort revealed a significant correlation between DV’s PI and atrial contraction with the onset of NEC. Our findings are consistent with a previous population-based study on preterm infants identifying AREDF and a high PI of the DV as risk factors for NEC and sepsis. This result highlights that both gut ischemia and sepsis are factors involved in the etiology of NEC [23].

Prematurity is well known to be associated with a high risk of developing morbidities, such as NEC [25]. In our sample, lower BW and abnormal findings on Doppler velocimetry of the umbilical artery emerged as the most common prenatal factors capable of predicting the onset of NEC [8,26].

In all published studies, BW is the main factor correlated with NEC in the prenatal period, along with Doppler velocimetry in the umbilical artery. Our previous study emphasized the role of FGR as a predominant NEC risk factor, without distinguishing cases by Doppler findings and BW percentile [22]. Only one study considered a fetal abdominal circumference lower than the 5th percentile, while others referred to the 10th percentile [7]. However, the authors did not state what types of curves they were referring to. Two neonatal growth curves were compared in the present study, considering the newborn’s sex and prematurity. Our data indicated that the Fenton curve was better than the IG21 curve for identifying fetuses with growth restriction that were likely to develop NEC.

Our statistical analysis also showed that normalizing data as z-scores could identify fetuses at a higher risk of NEC more effectively than multiples of the median (MoMs). This might be due to the Fenton growth charts offering a better assessment of population variance in preterm infants, enabling the newborn with FGR at higher risk of NEC to be identified more accurately. To the best of our knowledge, this report is the first to describe a correlation between a Fenton z-score < −1.62 and NEC risk.

Several factors are implicated in NEC pathogenesis, including intestinal barrier dysfunction, abnormal bacterial colonization, excessive inflammation, and ischemia due to vasoconstriction [27,28]. While these factors have been well characterized, their cause-effect relationships with the onset of NEC remain unclear. FGR fetuses use brain- and heart-sparing mechanisms that facilitate the redistribution of the blood flow to vital organs, thereby depleting the blood flow to the gastrointestinal tract. The subsequent protracted intestinal ischemia and reperfusion damage could trigger the inflammatory cascade, making the intestinal barrier more susceptible to penetration by bacteria. Evidence of intestinal blood flow instability has led some authors to suggest a role for the superior mesenteric artery waveform on Doppler ultrasound [29].

Moreover, WBC counts were lower in our newborns with FGR, who subsequently developed NEC than in controls. This may be related to a deficient immune status in these newborns, whose BWs were lower and who would be exposed to more significant morbidity and mortality. Similarly, Christensen et al. identified early neutropenia in newborns small for their GA as a risk factor for NEC onset [30].

Finally, the incidence of NEC, has been described as 6–10 times higher in exclusively formula-fed infants compared to the exclusively breastfed ones since preterm infant formula appears to alter the intestinal flora selecting potential pathogenic bacteria such as Clostridia and Proteobacteria. By contrast in our study there was not significant difference in enteral nutrition between the two groups. This may due to our institution policy, which consists in starting enteral nutrition with human milk (mother or donor source) as the first option in all preterm neonates.

### 4.3. Strengths and Limitations

This is the first study to have identified a significant role for DV pulsatility as a predictor of NEC, in addition to the well-known part played by an AREDF in the umbilical artery. Furthermore, our results also showed that the Fenton z-score was significantly more effective than the IG21 z-score in identifying newborns with FGR at risk of NEC. When IG21 or Fenton growth chart MoMs were considered, neither could predict NEC onset among our newborns with FGR.

The previously cited authors had only considered the 10th or 5th BW percentiles; here, we instead evaluated continuous BW values in terms of z-scores instead. We also examined some ROC curve models that considered the main obstetric and neonatal factors significantly associated with NEC. For the first time, WBC count and maternal parity were considered in addition to fetal Doppler findings, a BW below the third percentile, and postnatal conditions.

The main limitations of the present study lie in the retrospective design and small sample size.

## 5. Conclusions

Fetal Doppler findings proved effective in predicting NEC in FGR newborns, with the DV’s PI having the greatest predictive value. Including Doppler velocimetry of the DV in NEC prediction models might help to improve their specificity. Regarding fetal growth assessments, the Fenton z-score was highly sensitive in predicting NEC onset among FGR newborns.

## Figures and Tables

**Table 1:** Population description.

	Controls (29)	NEC (24)	*p*
Maternal characteristics and pregnancy management			
Maternal age (years)	34.00 (32.00–36.00)	36.50 (31.75–39.00)	0.299
Nulliparity	41.38% (12/29)	58.33% (14/24)	0.219
Medically assisted procreation	13.79% (4/29)	16.67% (4/24)	0.771
RDS prophylaxis	62.07% (18/29)	75.00% (18/24)	0.315
Delivery by CS	96.55% (28/29)	87.50% (21/24)	0.214
Pregnancy and fetal characteristics			
Pre-eclampsia	31.03% (9/29)	41.67% (10/24)	0.422
Gestational diabetes	3.45% (1/29)	4.17% (1/24)	0.891
Premature preterm rupture of membranes	13.79% (4/29)	12.50% (3/24)	0.890
Chorioamnionitis	6.90% (2/29)	8.33% (2/24)	0.844
Maternal Doppler			
Mean uterine arteries PI > 95th percentile	41.38% (12/29)	62.50% (15/24)	0.126
Bilateral uterine arteries notching	24.14% (7/29)	45.83% (11/24)	0.097
Fetal Doppler			
MCA PI< 5th percentile	41.38% (12/29)	54.17% (13/24)	0.353
UA AREDF	17.24% (5/29)	41.67% (10/24)	<0.05
DV PI > 95th percentile	10.34% (3/29)	41.67% (10/24)	<0.05

Abbreviation List: necrotizing enterocolitis (NEC); respiratory distress syndrome (RDS); Cesarean delivery (CS); middle cerebral arterial Doppler (MCA); absent or reversed end-diastolic flow (AREDF), ductus venosus Doppler (DV); umbilical artery (UA); pulsatility index (PI).

**Table 2:** Neonatal characteristics.

	Controls (29)	NEC (24)	*p*
Neonatal characteristics			
Neonatal male sex	51.72% (15/29)	66.67% (16/24)	0.272
Gestational age at delivery			
Days	213 (192–230)	196 (185–211)	0.100
Weeks	30.43 (27.43–32.86)	27.93 (26.39–30.18)	0.100
Apgar score at 5 min	8.00 (7.00–8.00)	8.00 (7.00–8.00)	0.857
Apgar score at 10 min	8.00 (8.00–9.00)	8.00 (8.00–9.00)	0.698
Cord blood pH	7.31 (7.28–7.34)	7.32 (7.28–7.35)	0.802
Birthweight (grams)	880.00 (650.00–1270.00)	747.50 (558.75–943.75)	0.186
Birthweight (Fenton z-score)	−1.66 (−2.74–1.16)	−3.09 (−4.21–1.88)	<0.05
Birthweight (Fenton MoM)	0.70 (0.63–0.77)	0.73 (0.64–0.78)	0.681
Birthweight (IG21 z-score)	−1.68 (−2.11–1.14)	−1.61 (−2.05–1.43)	0.639
Birthweight (IG21 MoM)	0.75 (0.65–0.82)	0.74 (0.65–0.90)	0.754
Fetal blood sample at birth			
Hb (g/L)	153.00 (145.00–170.00)	159.00 (143.00–168.50)	0.897
Platelets (×10^9^/L)	183.00 (133.00–225.00)	178.00 (107.50–224.00)	0.587
WBC (×10^9^/L)	6860.00 (5530.00–10,470.00)	4290.00 (2535.00–6360.00)	<0.05
Fetal cardio-pulmonary resuscitation	44.83% (13/29)	83.33% (20/24)	<0.05
Fetal tracheal intubation at birth	27.59% (8/29)	37.50% (9/24)	0.441
Fetal tracheal intubation after birth	3.45% (1/29)	0.00% (0/24)	0.358
Neonatal ventilation within the first 24 h of life			
1 Spontaneous breathing	24.14% (7/29)	16.67% (4/24)	0.504
2 Synchronized intermittent mandatory ventilation	41.38% (12/29)	70.83% (17/24)	<0.05
3 Nasal continuous positive airway pressure	10.34% (3/29)	8.33% (2/24)	0.803
4 High-flow nasal cannula oxygen	20.69% (6/29)	4.17% (1/24)	0.077
5 High-frequency oscillatory ventilation	3.45% (1/29)	0.00% (0/24)	0.358
Neonatal RDS	48.28% (14/29)	79.17% (19/24)	<0.05
Surfactant use	62.07% (18/29)	79.17% (19/24)	0.177
Apnea of prematurity	34.48% (10/29)	45.83% (11/24)	0.400
IVH	34.48% (10/29)	25.00% (6/24)	0.454
Neonatal sepsis	3.45% (1/29)	0.00% (0/24)	0.358
Respiratory acidosis	27.59% (8/29)	41.67% (10/24)	0.281
PDA	24.14% (7/29)	54.17% (13/24)	<0.05
PFO	34.48% (10/29)	45.83% (11/24)	0.400
Inotropic support	6.90% (2/29)	25.00% (6/24)	0.067
Enteral nutrition duration (days)	1.00 (1.00–1.00)	2.00 (1.00–3.00)	0.188
Type of Enteral nutrition HM (%)	26/29 (89.65)	20/24 (83.33%)	0.6881

Abbreviation List: necrotizing enterocolitis (NEC); intergrowth-21st (IG21); multiple of median (MoM); white blood cell (WBC); hemoglobin (Hb); respiratory distress syndrome (RDS); intraventricular hemorrhage (IVH); persistent patent ductus arteriosus (PDA); patent foramen ovale (PFO); human milk (HM).

**Table 3:** Predictivity of parameters according to nominal logistic regression followed by ROC analysis for univariate models.

	OR (*)	p (ŧ)	AUC (*)	Specificity (*) (**)	Sensitivity (*) (**)
Maternal and fetal Doppler (prenatal)					
Bilateral uterine artery notching	2.6593 (0.8256–8.5657)	0.101	61% (48–74%)	76% (50–100%)	46% (25–100%)
UA AREDF	3.4286 (0.9728–12.084)	0.055	62% (50–74%)	83% (66–97%)	42% (21–62%)
DV PI > 95th percentile	6.1905 (1.46–26.2484)	0.013	66% (54–77%)	90% (79–100%)	42% (21–62%)
Maternal and pregnancy characteristics					
Maternal age (years)	1.0468 (0.935–1.172)	0.427	58% (42–75%)	86% (66–97%)	46% (25–71%)
Nulliparity	1.9833 (0.6618–5.9438)	0.221	58% (45–72%)	59% (0–100%)	58% (0–100%)
Gestational age at delivery (days)	0.9833 (0.961–1.0061)	0.150	63% (48–79%)	52% (31–97%)	79% (29–96%)
Post-natal characteristics					
Fetal cardio-pulmonary resuscitation	6.15385 (1.67835–22.56368)	0.006	69% (57–81%)	55% (38–72%)	83% (67–96%)
PDA	3.71429 (1.15316–11.96363)	0.028	65% (52–78%)	76% (59–90%)	54% (33–75%)
Inotropic support	4.5 (0.81564–24.827)	0.084	59% (49–69%)	93% (79–100%)	25% (8–50%)
Birthweight (grams)	0.9993 (0.9981–1.0006)	0.289	61% (45–76%)	38% (28–97%)	88% (25–96%)
Birthweight (Fenton z-score)	0.6682 (0.4713–0.9474)	0.024	68% (54–83%)	48% (24–93%)	83% (42–100%)
Birthweight (Fenton z-score ≤ −1.62)	4.6667 (1.2753–17.0772)	0.020	66% (54–78%)	48% (31–66%)	83% (67–96%)
Newborn WBC (×10^9^/L)	0.9998 (0.9996–0.9999)	0.015	74% (60–88%)	79% (59–100%)	71% (38–92%)
Newborn WBC ≤ 5255 × 10^9^/L	9.3095 (2.6465–32.7479)	0.001	75% (63–87%)	79% (62–93%)	71% (50–88%)

(*) (95% CI); (**) Calculated considering the ROC curve best threshold of the model; (ŧ) The *p*-values indicate the statistical significance of each parameter considered, determined with Likelihood-ratio test. Abbreviation List: umbilical artery (UA); absent or reversed end-diastolic flow (AREDF), ductus venosus Doppler (DV); pulsatility index (PI); persistent patent ductus arteriosus (PDA); white blood cell (WBC).

**Table 4:** Predictivity of parameters according to nominal logistic regression followed by ROC analysis for multivariate models.

	OR (*)	*p* (ŧ)	AUC (*)	Specificity (*) (**)	Sensitivity (*) (**)
Model 1 (*p* < 0.05) (ŧŧ)			68% (55–81%)	90% (69–100%)	42% (21–71%)
UA AREDF	2.0569 (0.5089–8.3144)	0.312			
DV PI > 95th percentile	4.6982 (1.0161–21.7226)	0.048			
Model 2 (*p* < 0.05) (ŧŧ)			68% (54–83%)	41% (31–83%)	96% (62–100%)
Birthweight (Fenton z-score ≤ −1.62)	4.3918 (1.1861–16.2626)	0.027			
Birthweight (grams)	0.9995 (0.9982–1.0008)	0.469			
Model 3 (*p* < 0.05) (ŧŧ)			82% (71–94%)	76% (59–90%)	88% (71–100%)
Birthweight (Fenton z-score ≤ −1.62)	3.5051 (0.7672–16.0132)	0.106			
Newborn WBC ≤ 5255 × 10^9^/L	6.3635 (1.6871–24.0027)	0.006			
PDA	3.0366 (0.7747–11.9019)	0.111			
Model 4 (*p* < 0.05) (ŧŧ)			84% (72–95%)	72% (55–93%)	92% (71–100%)
Birthweight z-score Fenton ≤ −1.62	2.9388 (0.6127–14.0957)	0.178			
Newborn WBC ≤ 5255 × 10^9^/L	5.362 (1.3667–21.0366)	0.016			
PDA	2.8815 (0.7208–11.5184)	0.134			
DV PI > 95th percentile	2.3867 (0.4509–12.633)	0.306			

(*) (95% CI); (**) Calculated considering the ROC curve best threshold of the model; (ŧ) The *p*-values indicate the statistical significance of each parameter considered, determined with Likelihood-ratio test; (ŧŧ) This *p*-value is the significance for whole model determined with chi-squared test. Abbreviation List: umbilical artery (UA); absent or reversed end-diastolic flow (AREDF), ductus venosus Doppler (DV); pulsatility index (PI); white blood cell (WBC). Persistent patent ductus arteriosus (PDA).

## Data Availability

Not applicable.
